# Evaluation and Aggregation of Active Module Identification Algorithms

**DOI:** 10.1101/2025.10.06.680790

**Published:** 2025-10-07

**Authors:** Jason Liu, Min Xu, Jinchuan Xing

**Affiliations:** 1 Department of Genetics, Rutgers, The State University of New Jersey, Piscataway, NJ, USA.; 2 Human Genetics Institute of New Jersey, Rutgers, The State University of New Jersey, Piscataway, NJ, USA.; 3 Department of Statistics, Rutgers, The State University of New Jersey, Piscataway, NJ, USA.

## Abstract

**Background:**

High-throughput sequencing methods have generated vast amounts of genetic data for candidate gene studies. As a part of the analysis, candidate genes are often analyzed through Gene-Gene interaction (GGI) networks. These networks can become very large, necessitating efficient methods to reduce their complexity. Active Module Identification (AMI) is a common method to analyze GGI networks by identifying enriched subnetworks representing relevant biological processes. Multiple AMI algorithms have been developed for biological datasets, and a comprehensive assessment of these algorithms and a comparative analysis of their behaviors across a variety of use-cases are crucial for their appropriate applications.

**Results:**

In this study, we use the Empirical Pipeline (EMP) to evaluate four AMI algorithms – PAPER, DOMINO, FDRnet, and HotNet2 – on their ability to produce context-specific enrichment. When testing the algorithms on four biological datasets, our results reveal that no single algorithm outperforms the others across all datasets. Moreover, the output modules are often dissimilar, suggesting that different algorithms capture complementary biological signals. Our results suggest that a comprehensive analysis requires the aggregation of outputs from multiple algorithms. We propose two methods to this end: a spectral clustering approach for module aggregation, and an algorithm that combines modules with similar network structures called Greedy Conductance-based Merging (GCM).

**Conclusions:**

Overall, our results advance our understanding of AMI algorithms and how they should be applied. Tools and workflows developed in this study will facilitate researchers working with AMI algorithms to enhance their analyses. Our code is freely available at https://github.com/LiuJ0/AMI-Benchmark/.

## 1 Introduction

In recent years, high-throughput sequencing methods allow the production of large amounts of data for genetic research. Using the high-throughput data, the medical genetics field has advanced significantly in the understanding and treatment of many diseases and disorders, including hundreds of rare Mendelian disorders (Baxter et al.; 2022; Posey et al.; 2019), cancers (Swanson et al.; 2023), and complex diseases (Lowe and Reddy; 2015). Despite its success, current medical genetics research faces a major difficulty that impedes its full development: the complexity of the underlying genetic structure of the disease. That is, diseases often result from mutations in a large number of potential risk genes with a high degree of heterogeneity and interaction. Therefore, many disease-causing mutations and mutated genes are not present in all patients’ genomics data. Focusing solely on genes with the strongest evidence would result in a high number of false negatives, while relaxing the selection criteria could result in a high number of false positives, which could lead to unproductive downstream experiments and unnecessary expenditure of resources.

To further prioritize the candidate genes, one standard analysis in complex disease genetic studies is constructing a gene-gene interaction (GGI) network to account for the genetic heterogeneity (Reviewed in Wong et al. (2021)). The GGI network treats each candidate gene as a node, and uses available biological information from knowledge databases to assign edges to connect the genes. The constructed GGI network typically has a large number of edges even for a small number of candidate genes. Moreover, researchers often need to add intermediate nodes to the network to ensure that the network is connected, which further increases the size and complexity of the network. When the network becomes too complex, researchers often take additional manual approaches to reduce the size of the network, either by referencing existing results from literature or by using more stringent criteria for candidate node selection. However, these ad-hoc methods have serious drawbacks: the literature-based approach may be biased by the systemic over-representation of certain genes in the literature, while more stringent selection criteria might reduce the power of the candidate gene discovery.

To address this problem, a number of *active module identification* (AMI) algorithms have been developed (Reviewed in Levi et al. (2021) and Lazareva et al. (2021)). These algorithms are designed to identify connected subnetworks called *modules*, which represent biological processes or functional units. The modules provide additional evidence for biologically meaningful enrichment in GGI networks and prioritize candidate genes within the module for downstream analysis. However, these algorithms were developed using different clustering principles and some of them were developed for specific biological questions. A comprehensive assessment of these algorithms on real biological data is still lacking. In this study, we implemented and evaluated four AMI algorithms, and analyzed four empirical datasets using these algorithms. Then, we use the Earth Mover’s Distance as a way to determine the similarity between algorithms. We show that the methods produce complementary results with distinct module characteristics. Based on these findings, we developed a spectral clustering method to identify groups of genes that are consistently assigned to the same module by multiple methods. We also introduce the Greedy Merging by Conductance (GCM) algorithm, which leverages the network structure to effectively combine modules produced by different methods, enhancing the biological interpretability of the identified modules.

## 2 Results

### 2.1 Overview of AMI algorithms

**Figure 1:**
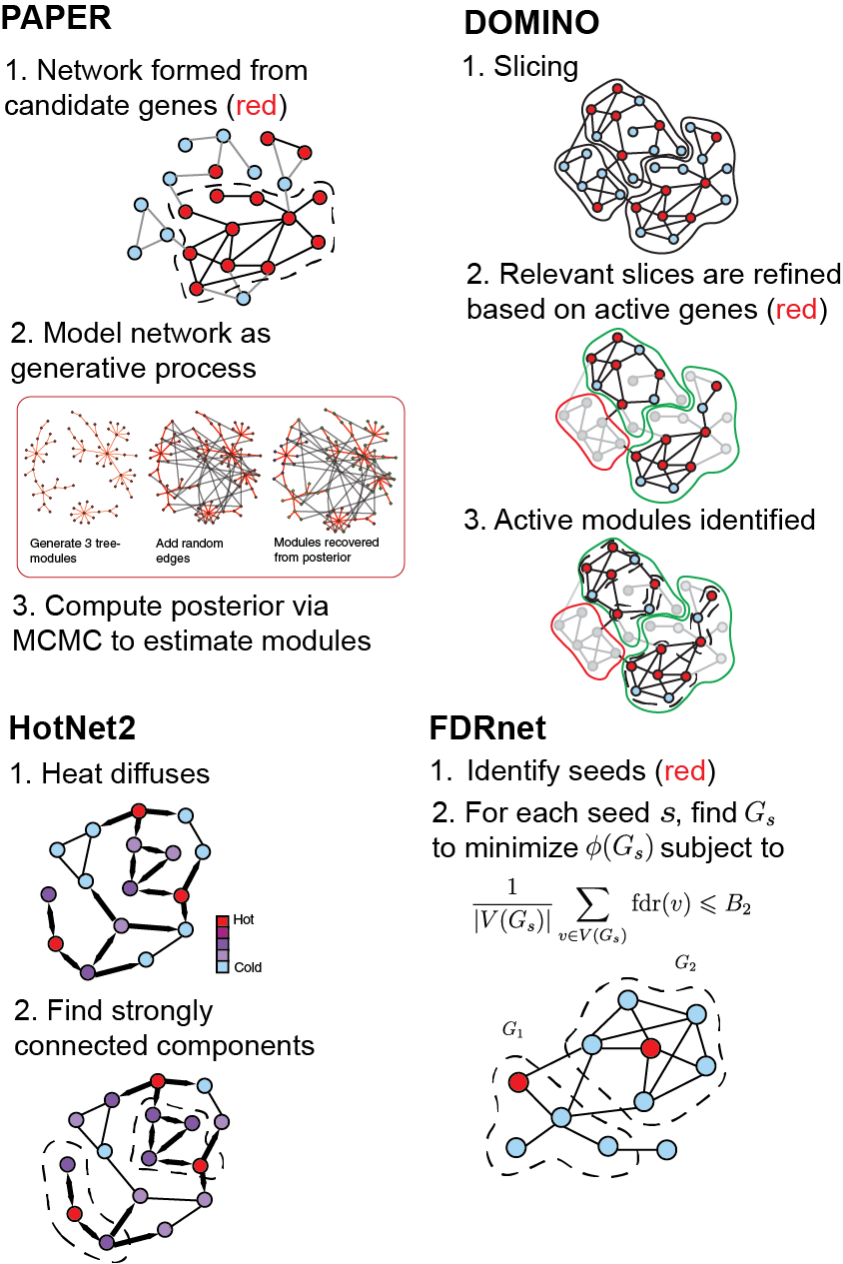
Overview of AMI algorithms.

**Figure 2:**
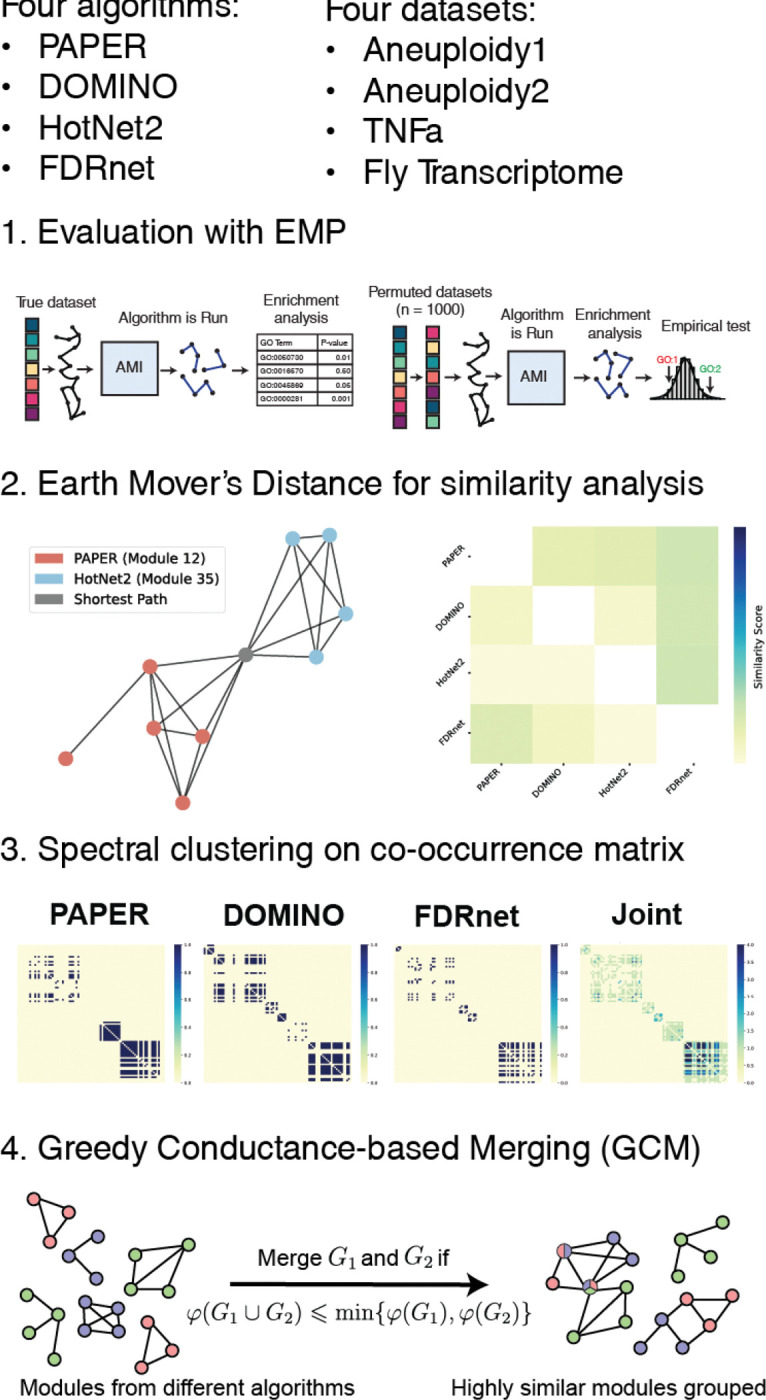
Project Workflow.

We study four algorithms for active module identification: PAPER (Crane and Xu; 2024), DOMINO (Levi et al.; 2021), HotNet2 (Leiserson et al.; 2015), and FDRnet (Yang et al.; 2021). We choose these four algorithms because they each have a distinct approach to the AMI problem and together cover a diverse spectrum of methodological paradigms: PAPER uses Bayesian modeling, DOMINO uses modularity minimization, HotNet2 uses network diffusion, and FDRnet uses constrained optimization. We briefly describe these algorithms below and in Figure 1. We give details on the implementation and parameter choices in the Methods and Materials section (Section 4). Detailed descriptions of the algorithms can be found in Additional File 1. The overall workflow of our evaluation approach, including the algorithm assessment, similarity analysis, and module aggregation steps, is illustrated in Figure 2.

- **PAPER** is a Bayesian method for detecting modules/communities on a general network. It models the observed network as being generated from a latent growth process. The growth process produces disjoint random tree-components according the preferential attachment mechanism and adds additional independent Erdős–Rényi random edges to ensure that the network is fully connected. The underlying disjoint trees partition the nodes into clusters and model the module structure.
  The disjoint tree-components are unobserved but PAPER uses Markov Chain Monte Carlo algorithms to sample from their posterior distribution given the observed network. Each MCMC sample is a plausible collection of modules and we aggregate them to output the final set of modules. We apply PAPER on the sub-network induced by the set of active genes.
- **DOMINO** is a coarse to fine modularity-based algorithm. Before seeing the AMI information, DOMINO first applies a preprocessing step where it partitions network into disjoint slices to improve computational efficiency. Once the AMI scores are obtained, DOMINO retains only those slices which contain a significant number of active genes. Each of the retained slices is further trimmed through a method based on prize collecting Steiner trees. Finally, DOMINO applies the Newman–Girvan modularity algorithm on the trimmed slices to produce a set of modules and retains only those modules deemed significant according to an over-representation test.
- **HotNet2** is based on a diffusion process where the initial AMI scores are represented as ”heat” and diffused through the interaction network via a random walk process with restart. This induces an “exchanged heat” matrix H. HotNet2 then defines a directed graph which contains an edge between node i and j if $H_{ij}$ is sufficiently large in magnitude. Finally, it outputs the strongly connected components of the directed graph as the output modules.
- **FDRnet** uses empirical Bayesian analysis to estimate local false-discovery rates (FDR) for a set of input genes. Genes with a local FDR less than the B parameter are considered seeds. For each seed, a random walk is conducted to compute a PageRank vector. The subnetwork identification problem is formulated as a mixed-integer linear programming problem, whose objective is to minimize the conductance (Definition 4) for the network while ensuring the FDR does not exceed the bound B.

### 2.2 Overview of the datasets

To evaluate the four algorithms, we selected four candidate gene datasets (Table 1) where the activity score of each gene represents either the significance of the mutation burden of a gene between the cases and the controls, or the significance of gene expression differences.

1. The dataset “**Aneuploidy1**” (Tyc et al.; 2021; Biswas et al.; 2024) contains data from whole exome sequencing (WES) of 178 patients undergoing in vitro fertilization (IVF) treatment, divided into high (n=93) and low (n=85) aneuploidy rate groups. Gene scores represent the significance of the variant burden in a gene in the high aneuploidy rate group compared to the low rate group, as determined using VAAST (Hu et al.; 2013).
2. The dataset “**Aneuploidy2**” (Tyc et al.; 2020) contains WES data from 160 patients undergoing IVF treatment, divided into high (n=68) and low (n=92) aneuploidy rate groups. Gene scores represent the significance of the variant burden in each gene in the high aneuploidy rate group compared to the low rate group, as determined using VAAST (Hu et al.; 2013).
3. The dataset “**TNFa**” (Schmidt et al.; 2015) contains RNA sequencing (RNA-seq) data from multiple cell types treated with tumor necrosis factor (TNF) for 90 minutes versus control. The gene scores represent the significance of the differential transcriptional activity between TNF-treated and vehicle control conditions.
4. The dataset “**Fly Transcriptome**” (Sun et al.; 2024) contains expression microarray data from different tissues of Drosophila melanogaster. Gene scores represent the significance of the expression of a gene in ovary versus other tissues.

We use three network databases chosen based on their diversity in size and node-edge density ratio (Table 2). For the Aneuploidy1 and Aneuploidy2 datasets, we take our network to be the union of the STRING (Szklarczyk et al.; 2017), GIANT(v2) (Greene et al.; 2015; Wong et al.; 2018), and ConsensusPathDB (Herwig et al.; 2016) human GGI networks, as described in our previous studies (Sun et al.; 2022; Biswas et al.; 2024) and we abbreviate the network as **SGC**. For the TNFa dataset, we use the Database of Interacting Proteins (which we abbreviate as **DIP**) network (Xenarios et al.; 2000) because of its small size, high-confidence edges, and high performance in a gene set recovery task (see Section 4.2 for more details). For the Drosophila analysis we use a high-confidence subnetwork of Drosophila GGI from STRING that contains only interactions with a confidence score over 900, which means that they are supported by strong experimental or database evidence. We refer to this network as **STRING**.

### 2.3 Evaluation of AMI algorithms

**Figure 3:**
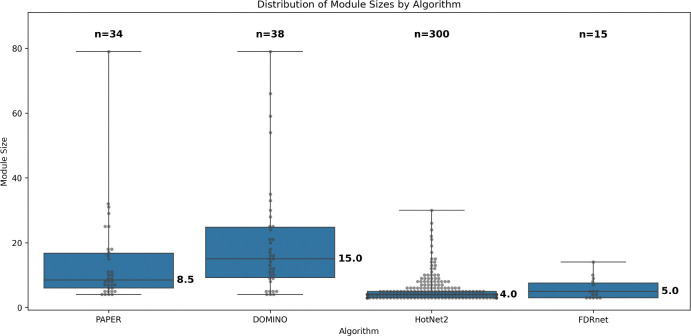
**Distribution of module sizes summed across all datasets. n** denotes total number of modules. Median is marked.

To systematically evaluate the AMI algorithms, we first compare their basic output characteristics across the datasets (Figure 3). We see that the different algorithms produce modules whose size distributions are quite different. PAPER and DOMINO tend to produce larger modules and have higher variation in modules sizes compared with HotNet2 and FDRnet. HotNet2 produces smaller modules (median size = 4) in much greater quantities (n = 300) than the other algorithms. FDRnet produces only 15 small modules (median size = 5) across all four datasets. The modules produced are recorded in Additional File 2: Table S1.

To evaluate the performance of the algorithms, we use the EMpirical Pipeline (EMP) framework proposed by Levi et al. (2021). First, we compute all the GO terms that pass a hypergeometric test with respect to each module. We refer to this set of GO terms as **HG terms**. Next, EMP performs a permutation-based null test where it permutes the gene activity scores in the dataset, runs the algorithm on the permuted datasets, and performs the same gene ontology enrichment analysis to generates a null distribution. We then compare the HG terms computed on the non-permuted real dataset against the null distribution, reject the ones whose empirical frequency in the null distribution is low, and refer to these as *empirically-validated* terms or **EV terms** (see step 1 of Figure 2).

We use four of the metrics given by Levi et al. (2021): the empirically-validated to hypergeometric ratio (EHR), biological richness, module-level EHR (mEHR), and intra-module homogeneity.

#### 2.3.1 Dataset Specificity

The EHR measures the specificity of an algorithm’s output for a given dataset; high values indicate that the algorithm’s output is more relevant to the dataset. It is defined as

$$EHR=\frac{\#(EV\;\text{terms}\;\text{across}\;\text{all}\;\text{modules})}{\#(HG\;\text{terms}\;\text{across}\;\text{all}\;\text{modules})}.$$

Overall, PAPER and FDRnet achieve the highest EHR across all datasets (Table 3).

#### 2.3.2 Module Quality

While EHR provides a cohesive metric for the performance of an algorithm, biological insights are derived from the inspection of individual modules. Therefore, we also compute the mEHR by restricting the EV and HG terms to the module level. That is, for a given module,

$$mEHR=\frac{\#(EV\;\text{terms}\;\text{enriched}\;\text{in}\;\text{the}\;\text{module})}{\#(HG\;\text{terms}\;\text{enriched}\;\text{in}\;\text{the}\;\text{module})}.$$

**Figure 4:**
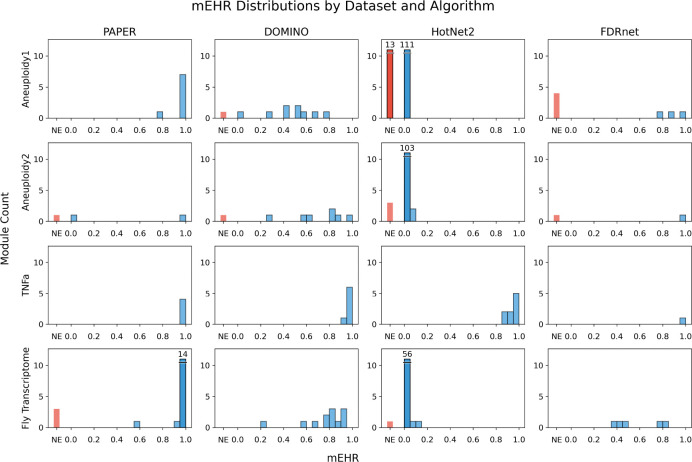
**mEHR distributions per algorithm and dataset.** “NE” (orange bar) stands for no enrichment. Slashed bar with number indicates overflow.

We present the distribution of mEHR values across all modules for each algorithm and dataset in figure 4. Modules in the SGC network often have lower mEHR enrichment, comparing to other networks.

#### 2.3.3 Functional Diversity

Biological richness measures the number of non-redundant GO terms in the algorithm’s output. Due to the hierarchical nature of the gene ontology, many groups of genes are enriched with similar GO terms, resulting in a large number of HG terms, which may not accurately reflect the diversity in the algorithm’s output. DOMINO has the highest number of EV GO terms as well as non-redundant GO terms (i.e. biological richness) among the four algorithms (Table 4). The interpretation of biological richness as a measure can depend on the context: while high richness could indicate both a relevant diversity in enrichment, it could also indicate a large number of unrelated GO terms due to ineffective handling of noise. The redundancy is measured by the *Resnik similarity* (Resnik; 2011) and described further in Section 4.3.1.

#### 2.3.4 Functional Coherence

An ideal AMI algorithm should identify individual modules that are enriched for distinct biological functions. Therefore, different biological processes can be identified based on the modules.

While it is desirable for the enrichment of a module to be specific to the dataset, the enrichment of the module should also be different from the others to make biological interpretation easier. The intra-module homogeneity measures how homogeneous the EV terms for each module are. There is no single algorithm that outperforms the others for this metric, though DOMINO has the highest intra-module homogeneity averaged across the datasets (2.539)(Table 5). This criteria also uses the Resnik similarity as a measure for how different modules are, and is described in detail in Section 4.3.1.

### 2.4 AMI algorithms produce distinct outputs

Our evaluation showed that multiple algorithms performed well on any given dataset but no single algorithm performed the best across all the datasets. Therefore, it is important to assess the heterogeneity of the outputs of the different algorithms so that practitioners can understand whether high-performing algorithms identify similar or complementary signals.

#### 2.4.1 Earth mover’s distance

To quantify the similarity between modules identified by the different algorithms, we use the earth mover’s distance (EMD). The EMD is a way of measuring the distance between a pair of modules that take into account their distance on the network; it is thus more informative than the naive approach of computing the number of genes that overlap in the two modules. Intuitively, it is the minimum amount of work needed to move a unit of mass/earth (uniformly distributed on the genes) from one module to the other so that the genes of the other module are covered uniformly.

##### Definition 1 (Earth mover’s distance).

Let 𝒢 be the global GGI network. Let ${dist(v}_{i},v_{j})$ be the distance of the shortest path between nodes $v_{i},v_{j}\in V(𝒢)$. Given modules $M_{1}$ and $M_{2}$ with $n_{1}$ and $n_{2}$ nodes, respectively, we say $C\in [0,\infty {)}^{n_{1}\times n_{2}}$ is a flow matrix if the row-sum of each row of C is $\frac{1}{n_{1}}$ and the column-sum of each column of C is $\frac{1}{n_{2}}$. Then, we define the earth mover’s distance between modules $M_{1}$ and $M_{2}$ to be

$$d_{EM}\left(M_{1},M_{2}\right)=\underset{C\;\text{flow}\;\text{matrix}\;}{min}\sum_{i=1}^{n_{1}}\sum_{j=1}^{n_{2}}C_{ij}\cdot dist\left(v_{i},v_{j}\right).$$

Intuitively, entry $C_{ij}$ is the amount of mass that flows from node i to node j, so the EMD is small when modules share genes that are close in the network, and large when the genes in the modules are far apart on the network. Therefore, it captures the similarity of modules, even when they share little or no overlap.

**Figure 5:**
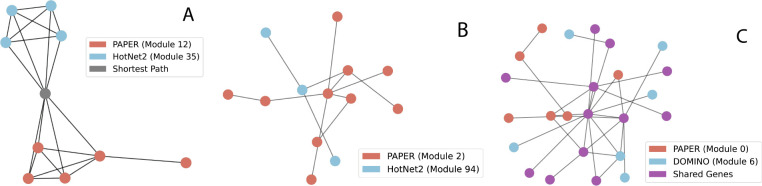
**Similar modules identified by different algorithms.** A: PAPER module and HotNet2 module in the Fly Transcriptome dataset, B: PAPER module and HotNet2 module in the Aneuploidy1 dataset, C: PAPER module and DOMINO module in the TNFa dataset.

By computing the pairwise EMDs between modules, we can identify similar modules. In the fly transcriptome dataset, we identified two modules with EMD 2.2 that are not overlapping and not even adjacent, but still close to one another (separated by one gene, Figure 5A). In the Aneuploidy1 dataset we identify two modules with EMD 2.214 that are adjacent (Figure 5B). In the TNFa dataset we identify two modules with multiple overlapping genes with an EMD of 0.529 (Figure 5C). Gene names for each module can be found in Additional File 2: Table S1.

Computing similar modules can also uncover *hidden* genes, which are biologically relevant but do not appear in modules or datasets. For example, in Figure 5A, we identify *Chrac-14* (Chromatin accessibility complex 14kD protein, colored in gray) as the shortest path between the two modules. Notably, *Chrac-14* is not present in the Fly Transcriptome dataset. In section 3.2, we address the biological significance of this discovery.

To compare the similarity between the two sets of modules outputted by two different algorithms, we propose two approaches of extending the EMD: one based on matching and one based on sums of minimums. The matching similarity, given in Definition 2, computes a one-to-one matching between the two sets of modules (from the smaller set to the larger set) such that sum of the EMDs between the matched pairs is minimized. We then take the sums of the EMDs and apply the function $x\mapsto \frac{1}{1+x}$ obtain a similarity measure that takes value in [0,1]. Two identical sets of modules would have a matching similarity of 1. We give the mathematical formulation below:

##### Definition 2 (Matching Similarity).

Let 𝒢 be the global GGI network, and two sets of modules ${ℳ}^{1}=\left\{M_{1}^{1},\ldots ,M_{n}^{1}\right\}$ and ${ℳ}^{2}=\left\{M_{1}^{2},\ldots ,M_{m}^{2}\right\}$, where we assume without loss of generality that n⩽m. Define $S_{n,m}$ to be the set of one-to-one mappings from {1,2,…,n} to {1,2,…,m}. The *matching similarity* between ${ℳ}^{1}$ and ${ℳ}^{2}$ is then

$${sim}_{M}\left({ℳ}^{1},{ℳ}^{2}\right):={\left(1+\underset{\tau \in S_{n,m}}{min}\;\sum_{j=1}^{n}\;{\;d}_{EM}(M_{j}^{1},M_{\tau (j)}^{2})\right)}^{-1}.$$

**Figure 6:**
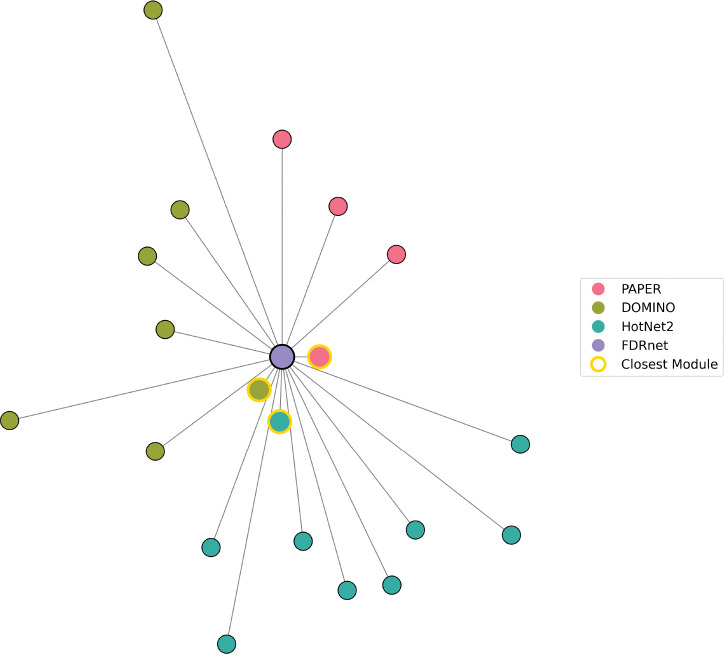
**Distances of all modules from FDRnet module.** Every algorithm has one module very close to the FDRnet module (highlighted in yellow), which makes the similarity defined by Definition 2 very small. Meanwhile, the other modules are very far apart, decreasing the similarity defined by Definition 3.

One drawback of matching similarity is that it can be misleading when one algorithm outputs many more modules than the other. For example, in the TNFa dataset, FDRnet produces only a single module that has small EMD to one module in PAPER, one module in DOMINO, and one more in HotNet2; see Figure 6. As a result, the matching similarity between FDRnet and all the other algorithms is close to 1 even though the other algorithms output many more modules. We therefore also propose a similarity measure using sums of minimum distances (Definition 3). Here, we sum the minimum EMD from each module in one set to any of the modules in the other set to give another notion of distance between the two sets of modules. We then convert it into a similarity measure via the mapping $x\mapsto \frac{1}{1+x}$ in the same way as the before.

##### Definition 3 (Sum Similarity).

Let 𝒢 be the global GGI network, and two sets of modules ${ℳ}^{1}=\left\{M_{1}^{1},\ldots ,M_{n}^{1}\right\},\;{ℳ}^{2}=\left\{M_{1}^{2},\ldots ,M_{m}^{2}\right\}$. The *sum similarity* between ${ℳ}^{1}$ and ${ℳ}^{2}$ is

$${sim}_{S}\left({ℳ}^{1},{ℳ}^{2}\right)\;:={\left(1+\frac{1}{2}\left\{\sum_{j=1}^{n}\;\underset{M\in {ℳ}^{2}}{min}\;{\;d}_{EM}\left(M_{j}^{1},M\right)+\sum_{k=1}^{m}\;\underset{M\in {ℳ}^{1}}{min}\;{\;d}_{EM}\left(M_{k}^{2},M\right)\right\}\right)}^{-1}.$$

**Figure 7:**
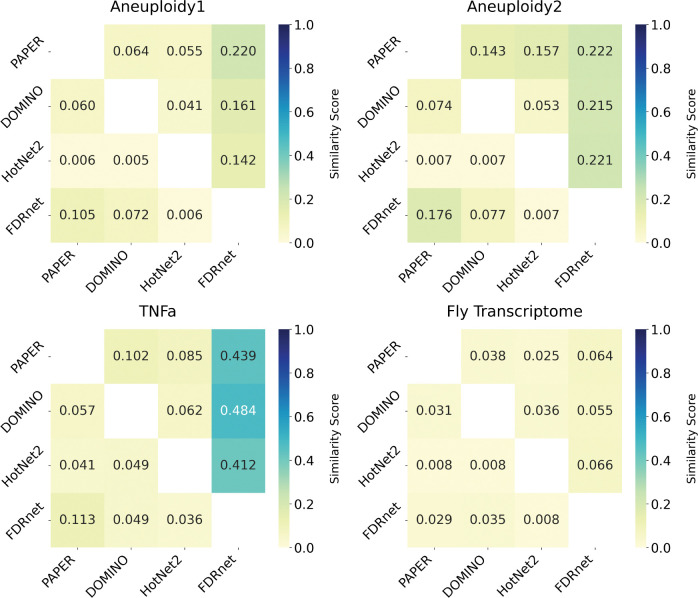
**Heatmap representations of similarity matrices for each dataset.** Darker colors indicate higher similarity. The upper diagonal represents the matching similarity (Definition 2) and the lower diagonal represents the sum similarity (Definition 3).

We computed the algorithm similarity for each of the datasets using both algorithms and present the results in Figure 7. The average matching-based similarity score (averaged across all pairs of algorithms) is 0.148 and the average sum-based similarity score is 0.033, which suggests that overall outputs of the algorithms are distinct although they may share a few similar modules.

### 2.5 Combining results across algorithms

Our similarity analysis suggests that while multiple algorithms may perform well on a dataset, they tend to identify distinct biological signals. Therefore, practitioners should use multiple algorithms in their analysis to capture the biological signal more completely. To facilitate the integration of results from multiple algorithms, we introduce two complementary methods for combining their outputs.

#### 2.5.1 Module aggregation with spectral clustering

First, we introduce a spectral clustering method that identifies groups of genes consistently assigned to the same module across multiple algorithms.

Given sets of modules ${ℳ}^{1},\ldots ,{ℳ}^{K}$, let $V=\cup_{k=1}^{K}\cup_{M\in {ℳ}^{k}}M$ be the set of unique genes that appear in them; we write n:=|V| and $V=\left\{v_{1},\ldots ,v_{n}\right\}$ using an arbitrary ordering on the genes. We define the n by n gene co-occurrence matrix C entrywise for genes $v_{i}$ and $v_{j}$ by

$$C_{ij}=\sum_{k=1}^{K}𝟙\{\exists M\in {ℳ}^{k}:v_{i},v_{j}\in M\}.$$

In other words, $C_{ij}$ is the number of times $v_{i}$ and $v_{j}$ are assigned to the same module. We performed spectral clustering using this matrix, more details of the method are provided in Section 4.6.

In our analysis, we excluded HotNet2 because of its weak performance in terms of EHR, and also because its module output is very disjoint from the rest: out of the 300 modules outputted by HotNet2, 239 (≈79.7%) share no overlap from any module with any other algorithm. We also excluded any modules disjoint from every other module. The clustering resulted in 23 clusters for the Aneuploidy1 dataset, 8 clusters for the Aneuploidy2 dataset, 4 clusters for the TNFa dataset, and 13 clusters for the Fly Transcriptome dataset.

**Figure 8:**
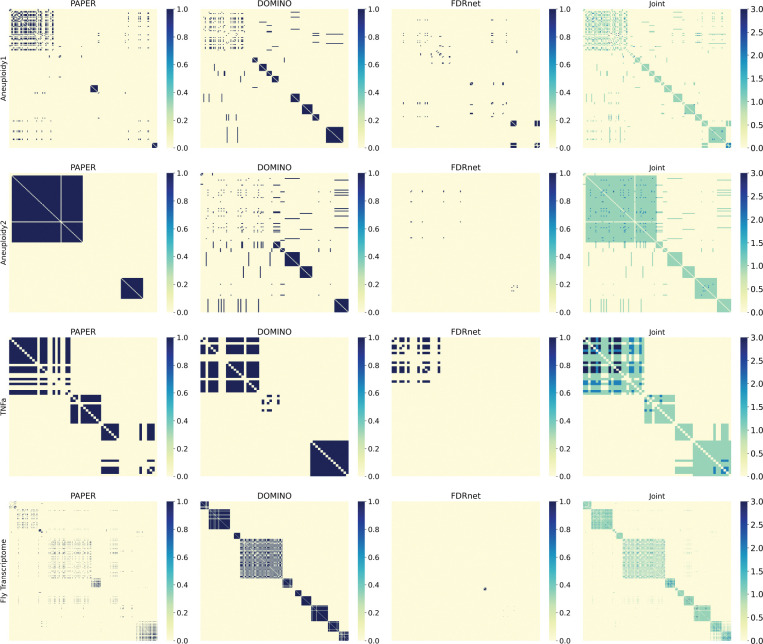
**Heatmap representations of module aggregation.** The first three columns each represent the individual co-occurence matrices of PAPER, DOMINO, and FDRnet respectively. The fourth column is the sum of the heatmaps. Notably, there is a cluster made up of modules from all three algorithms in the TNFa dataset.

**Figure 9:**
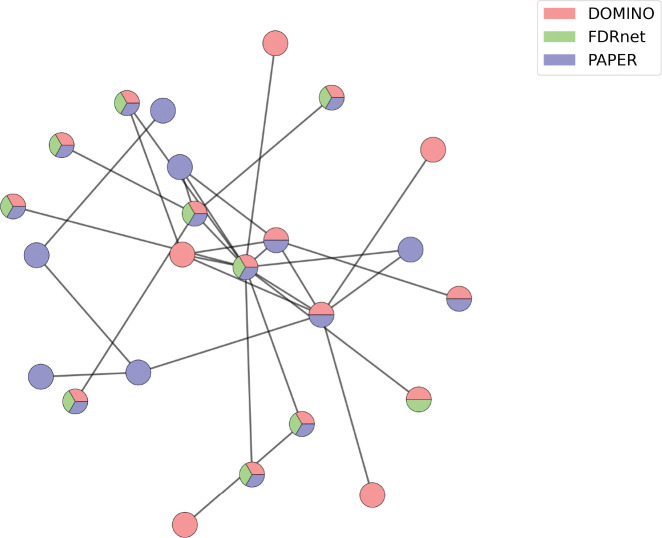
**Module cluster in the TNFa dataset,** identified by the module aggregation method, as shown in figure 8. Genes with multiple colors belong to multiple modules. Notably, this cluster is not identified by the conductance merging algorithm (Algorithm 1).

The resulting clusters (Figure 8) reveal algorithmic-specific and consensus modules depending on the dataset. In particular, the Aneuploidy1 and Aneuploidy2 datasets contain 9 and 6 clusters corresponding to unmerged modules, respectively, whereas in the TNFa and Fly Transcriptome dataset, there is only one cluster in each corresponding to an unmerged module. Moreover, one cluster in the TNFa dataset shows significant consensus between all three algorithms (Figure 9).

#### 2.5.2 Module merging using conductance

One drawback of spectral clustering is that it is not very informative if there is very little overlap to begin with (see Aneuploidy1, Aneuploidy2, and Fly Transcriptome in Figure 8). However, modules can be biologically related even without direct overlap. To address this limitation, we introduce the Greedy Conductance-based Merging (**GCM**) algorithm. GCM greedily merges modules based on their *conductance*, which measures how well-formed a given module is. The definition follows:

##### Definition 4.

The *conductance* of a module M is

$$\varphi \left(M\right)\;:=\frac{c}{2m\;+\;c},$$

where c is the number of edges on the boundary of M, and m is the number of edges in the interior of M. The conductance of a module is minimized when m is large and c is small, a desirable quality of modules.

##### Definition 5.

The *conductance ratio* between two modules $M_{1}$ and $M_{2}$ is

$$\rho \left(M_{1},M_{2}\right)\;:=\frac{\varphi \left(M_{1}\;\cup \;M_{2}\right)}{min\left\{\varphi \left(M_{1}\right),\;\varphi \left(M_{2}\right)\right\}}.$$

We choose to merge modules $M_{1}$ and $M_{2}$ when $\rho \left(M_{1},M_{2}\right)⩽1$ so that

$$\varphi \left(M_{1}\cup M_{2}\right)⩽min\left\{\varphi \left(M_{1}\right),\varphi \left(M_{2}\right)\right\},$$

meaning the resultant module is at least as well-contained as the better of the two original modules. Note that modules need not have any overlap at all to have a conductance ratio less than 1; thus, such a merging condition can be thought of as weaker than using direct overlap.

At each iteration of the GCM algorithm, we find a suitable pair of modules $M_{i^{*}}^{k^{*}}$ and $M_{\ell^{*}}^{j^{*}}$ that minimizes the conductance ratio. Then, we merge them and remove the merged modules from consideration in the next iteration. However, we allow merged modules (already containing other component modules) to be merged again with modules from algorithms not already contained in the module. On line 12, we set the conductance ratio of all modules contained in algorithms already merged to ∞, forbidding two modules from the same algorithm to be merged. This ensures that the merged output ${ℳ}^{*}$ has several desirable properties:

1. Every module in the input appears exactly once, either by itself or as a component in a merged module. Specifically, if M is an input module then $M\subset M^{*}$ for exactly one $M^{*}\in {ℳ}^{*}$.
2. Each module in ${ℳ}^{*}$ contains at most one module from any given algorithm, i.e. for any $M^{*}\in {ℳ}^{*}$ and $M_{1},M_{2}$ input modules from the same algorithm, we have $M_{1}\subset M^{*}$ implies $M_{2}\cap M^{*}=\emptyset$.

**Figure 10:**
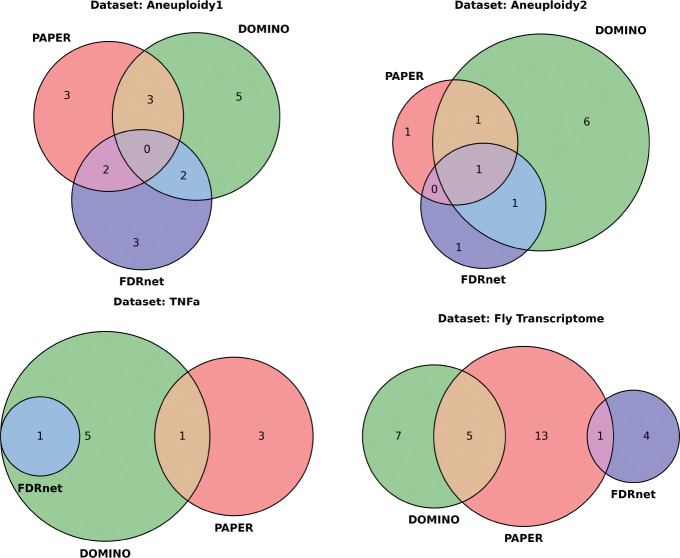
**Venn diagrams to visualize GCM.** Overlap indicates modules merged together.

After running GCM on the resultant modules of each of the algorithms, we identify several merged modules (Figure 10). Notably, we group module 6 of DOMINO and module 0 of FDRnet as in the module aggregation procedure, but we do not group module 0 of PAPER with it (Additional File 3: Table S2). We also identify more clusters (Aneuploidy1: 8, Aneuploidy2: 3, TNFa: 2, Fly transcriptome: 6) despite the lack of overlap of genes.

## 3 Discussion

AMI algorithms are fundamentally important to the analysis of biological networks, but their comparative performance, especially for algorithms based on different design principles, is still poorly understood. In this study, we systematically evaluated the performance of four AMI algorithms - PAPER, DOMINO, HotNet2, and FDRnet - across diverse biological datasets and network types. These four recent algorithms used four different types of module identification approaches: including Bayesian modeling (PAPER), modularity minimization (DOMINO), network diffusion (HotNet2), and constrained optimization (FDRnet). Our analysis reveals that PAPER and FDRnet generally have the highest EHRs, indicating a superior specificity to the underlying biological context. However, for each dataset there are multiple high-performing algorithms in terms of specificity. Our application of the Earth Mover’s Distance shows that algorithms play complementary roles, suggesting that the use of multiple algorithms for a given dataset may be beneficial. Finally, to streamline the joint analysis of multiple algorithms, we introduce a conductance merging algorithm that merges modules with similar network structures.

### 3.1 High-performing algorithms produce different outputs

Better performing algorithms in the sense of EHR, i.e. specificity, did not necessarily produce biologically similar modules when using the EMD as a metric of similarity (Figure 7). For example, in the TNFa dataset, PAPER, DOMINO, and HotNet2 achieve EHRs of 1, 0.987, and 0.976 respectively, but display very low pairwise similarity (average: 0.049 sum of EMDs, 0.083 matching). That is, all three algorithms produce valid enrichment specific to the dataset, but they represent distinct biological processes.

This result suggests that while there are multiple algorithms that produce dataset-specific solutions, no single algorithm can cover all biologically relevant modules. Therefore, using multiple high-performing algorithms is necessary to uncovering the full biological insight. This challenges current literature and common practice, which often recommends the use of a single “best” algorithm.

### 3.2 Computing similar modules using EMD can uncover hidden genes

Determining similar modules can aid the biological interpretation of them. For example, the module pair in Figure 5A play complementary roles in chromatin regulation. In the PAPER module, *Ada2b* and *WDA* are parts of the SAGA (Spt-Ada-Gcn5-Acetyltransferase) complex that contribute to histone acetylation (Soffers et al.; 2021). *D12* also plays a role as part of the ATAC complex, which stimulates nucleosome sliding (Suganuma et al.; 2012). Together, they act as a mechanism to promote gene expression. Meanwhile, the HotNet2 module contains *ACF*, which contributes to the establishment of a ground state of chromatin (Scacchetti et al.; 2018) as well as *CtBP* which acts as a transcriptional corepressor (Chinnadurai; 2000–2013). As a module, they establish repressive chromatin states.

One interesting aspect of identifying similar modules is that it can also reveal relevant genes not present in the modules. For example, for modules in Figure 5A, *Chrac-14* (shown in gray) acts as the shortest path between the PAPER and HotNet2 modules. Notably, *Chrac-14* is not present in the Fly Transcriptome dataset (it appears only in the STRING network). Therefore, it acts as a *hidden* gene: it was not directly implicated by the original experimental data but is revealed through the network topology to potentially play an important role in connecting functional modules. Functionally, *Chrac-14* participates in DNA repair and chromosomal DNA replication. As a member of the chromatin accessibility complex, it facilitates nucleosome sliding, which plays a role in DNA damage response (FlyBase; 2019). Therefore, it plays an important role in accurate chromosome segregation. Errors in chromosome segregation are shown to be a major cause of aneuploidy (Smith and Nambiar; 2020).

### 3.3 Different grouping methods can be used in different scenarios

The spectral aggregation and conductance merging algorithms provide alternative approaches to module grouping that are valuable in different contexts. When the modules in consideration have very little overlap, the spectral clustering algorithm can be uninformative (see Figure 8 for Aneuploidy1, Aneuploidy2, and Fly Transcriptome datasets). However, since the merging algorithm permits merges of neighboring modules rather instead of overlap, it can still give informative results. On the other hand, spectral aggregation can be more informative when there is greater overlap: the module cluster shown in Figure 9 is not identified by the merging algorithm (Figure 9). Researchers should therefore select the spectral clustering algorithm if there is very high overlap, and the merging algorithm if this condition does not hold.

### 3.4 FDRnet favors statistical significance over network structure

Both FDRnet and the conductance merging algorithm seek to optimize the conductance of the participant modules. In particular, we note that the merging algorithm also merges the modules of FDRnet. This means the merging algorithm introduces genes to the FDRnet modules that improve its conductance (and therefore modular structure), but that make the average FDR of the module too high to include. This suggests that FDRnet’s strict adherence to FDR thresholds may come at the cost of excluding structurally relevant genes.

### 3.5 Merging algorithms could help overcome parameter selection

A significant challenge in using AMI algorithms is parameter selection. For example, FDRnet depends primarily on a B parameter that controls the local FDR threshold of the modules produced. Even though this parameter can greatly influence the output modules, its selection can be arbitrary. Selecting optimal parameters is often done empirically through trial and error, which can be time-consuming to analyze.

The conductance merging (Algorithm 1) and spectral aggregation method (Section 2.5.1) we propose can be used to overcome this issue. We suggest running the same algorithm with different parameters, and then using these methods to consolidate the outputs together. This would reduce the dependency on parameter tuning, since modules that appear consistently across parameter settings are more likely to represent genuine biological signals. It also provides a measure of confidence in the identified modules, since those that emerge from a wide range of parameter settings can be considered more reliable. Future work could explore the usage of these methods to further overcome parameter selection.

### 3.6 Comparisons and Limitations

Previous works that introduce an AMI algorithm usually demonstrate that the algorithm outperforms other algorithms (e.g., Levi et al. (2021); Yang et al. (2021)). Our comparisons using multiple algorithms and multiple dataset suggests that the AMI algorithms have distinct behaviors depending on the underlying logic and assumptions. More detailed similarity analysis and aggregation are needed to determine the performance of the algorithms.

Some studies (e.g., Boyd et al. (2023); Pasquier et al. (2023)) use strict overlap between modules as a way to determine the similarity of the algorithms. This method is not informative between modules that do not overlap, since they can be very close or very far apart. We use the EMD as a way to improve on this analysis, since the EMD is more sensitive to the overall structure of the network and provides useful information even when the modules are disjoint. Furthermore, to our knowledge, there are no studies that introduce ways to aggregate the results of different outputs. We provide a greedy aggregation method for researchers who run multiple AMI algorithms and prefer one set of final modules for downstream analyses.

Our study has a few limitations. One limitation of this study is that each dataset is run only on a single GGI network. Previous studies show that many AMI algorithms do not learn from the input biological network, since they are equally capable of producing biologically relevant modules, even on random networks with the same degree distribution (Lazareva et al.; 2021). One way to control for this is to run each dataset on multiple networks, and do a cross-network comparison. On the other hand, the performance of the algorithms on the TNFa dataset (Table 3) seems to suggest that its higher-confidence edges lead to more dataset-specific results, which is counter to what is proposed in the study.

We were also unable to fine-tune the parameters of all the algorithms analyzed due to the long runtimes of EMP. This could potentially bias algorithms that are less sensitive to tuning or have ways to estimate parameters, such as HotNet2, as opposed to those that have arbitrarily selected parameters such as FDRnet.

Furthermore, our analysis primarily focuses on network topology as opposed to enrichment. We choose to do this because it is more informative for purposes other than enrichment, such as the identification of hidden genes or other important modules. Future studies could address this and provide a comprehensive analysis of similarity in terms of GO enrichment.

### 3.7 Conclusions and Future Directions

Moving forward, the module merging algorithm we develop can be used to analyze the outputs of any community detection or AMI algorithms on a given dataset. Since it only takes the full network and resultant modules as an input, its application is not limited to biological networks. To allow for further testing and more detailed investigation, we made our code is freely available at https://github.com/LiuJ0/AMI-Benchmark/.

In conclusion, our evaluation of AMI algorithms reveals their complementary nature in the GGI network analysis. Our application of the EMD suggests that many high-performing algorithms capture different biological signals, and that multiple methods should be used to create a more complete picture. To integrate the results, we propose a conductance merging algorithm that offers a principled approach to combining outputs of these different methods. These contributions advance our understanding of AMI algorithms and how they should be used, and provides practical tools to further their analysis.

## 4 Methods and Materials

### 4.1 Datasets

Candidate genes and associated p-values from datasets **Aneuploidy1** (Biswas et al.; 2024), **Aneuploidy2**, (Tyc et al.; 2020), and **TNFa** (Madsen et al.; 2015) were obtained from previous studies, respectively. For **Fly Transcriptome**, gene expression profiles in fly tissues were obtained from http://flyatlas.org/data.html, (Chintapalli et al.; 2007). Unknown, non-protein coding, RNA, and ribosomal proteins were removed from the gene set. Enrichment values for the ovary were calculated as the ratio of ovary-specific expression to the mean expression across all tissues. Statistical significance was assessed using a one-tailed t-test to identify genes upregulated in the ovary. Multiple-testing correction was applied through Bonferroni adjustment of p-values. Genes with adjusted p-value < 0.05 are considered significant.

### 4.2 Networks

#### SGC:

STRING integrates multiple data types including experimental data, pathway databases, and literature co-occurrence to create confidence-scored functional interactions. GIANT(v2) provides tissue-specific functional networks, while ConsensusPathDB integrates interaction data from 31 public databases to provide a comprehensive interaction landscape. For the network construction, we integrated and filtered interactions from all three databases using previously established methods (Sun et al.; 2022; Cao et al.; 2021). The network was built using custom Python software available at https://github.com/JXing-Lab/network-ppi.

#### DIP:

The second network is the Database of Interacting Proteins (DIP), which is much smaller than the other networks (with 3,000 genes and 5,000 interactions) but maintains a higher confidence of edges. Specifically, it provides the highest value per interaction when correcting for network size (Huang et al.; 2018). The *value* is defined to be the network’s improvement in the *gene set recovery task* compared to random networks of the same degree distribution, where a subset of genes is selected from a disease-associated gene set, and network propagation (see HotNet2 algorithm) is used to recover the ground-truth set. The network was retrieved from https://dip.doe-mbi.ucla.edu/dip.

#### STRING (fly):

The Drosophila melanogaster network was derived from the STRING database (version 12), which contains 5,033,960 total interactions. Each interaction is scored between 0 and 999 according to a systematic benchmarking process against KEGG pathway maps. Note that each score does not indicate the strength of the interaction, but instead confidence. We select edges with a score above 900, meaning rougly 1 out of 10 interactions might be incorrect (Szklarczyk et al.; 2017).

### 4.3 EMP

To evaluate the algorithms using EMP, we implemented each algorithm as a class directly usable for EMP; it is accessible at https://github.com/LiuJ0/AMI-Benchmark. We generate 1000 permutations for each algorithm and dataset. The EMP code was retrieved from https://github.com/Shamir-Lab/EMP.

#### 4.3.1 Evaluation Metrics

##### Biological Richness:

This is the number of non-redundant GO terms in the algorithm’s output. Let 𝒢=(V,E) be the global GGI network. Let 𝒞 be a collection of GO terms, and A:𝒞→𝒫(V) associate each GO term with its gene annotations. Note 𝒞 has the structure of a directed acyclic graph under the “is a” relation; inducing a parent-child relationship. Then, the *Resnik similarity* (Resnik; 2011) of $c_{1},c_{2}\in 𝒞$ is

$${sim}_{R}\left(c_{1},c_{2}\right)\;:=\underset{c\;\text{common}\;\text{parent}\;\text{of}\;c_{1},c_{2}}{max}\;-log\left(\frac{\left|A(c)\right|}{\left|V\right|}\right).$$

Redundant GO terms based on the Resnik similarity are filtered out according to the method described in Supek et al. (2011).

##### Intra-module Homogeneity:

Given a set of GO terms ℰ⊂𝒞 enriched in a set of modules ℳ and a similarity threshold α, construct a graph T on ℰ such that $c_{i}c_{j}\in E(T)$ if and only if ${sim}_{R}\left(c_{i},c_{j}\right)⩾\alpha$. Write D(H) to be the edge density of H as a subgraph of T. Then, the *intra-module homogeneity* of ℳ is

$$\frac{1}{\left|ℳ\right|}\sum_{M\in ℳ}\frac{D(M)}{D(T)}.$$

Following the work of Levi et al. (2021), we set α:=4 in table 5.

### 4.4 Algorithm implementations

We implemented the algorithms as follows:

#### PAPER:

PAPER was originally implemented as a community detection algorithm (i.e., for networks without weights). To implement it as an AMI algorithm, we also weigh the edges by the rule $e\left(v_{i}v_{j}\right)=\sqrt{v\left(v_{i}\right)v\left(v_{j}\right)}$ where v:V(𝒢)→[0,1] is the node weight, and $v_{i},v_{j}\in V(𝒢)$. We construct a subnetwork using the genes and edges with a weight less than some parameter (typically 0.05). Then, we identify the largest connected component and run PAPER on it. On the true dataset, we run PAPER with parameters M=5000, Burn=1000. Because it is expensive to use such resources for the computation of each permutation, we use parameters M=400, Burn=10 for the permutations. In both cases, we set size_thresh=0.02, birth_thresh=0.6. Parameters were tuned empirically. Modules of size smaller than 3 were discarded to be consistent with the other algorithms. Code was retrieved from https://github.com/nineisprime/PAPER.

#### DOMINO:

We run DOMINO with its default parameters (slice_threshold=0.3, module_threshold=0.05). Code was retrieved from https://github.com/Shamir-Lab/DOMINO.

#### HotNet2:

HotNet2 depends on δ and β parameters. We used the provided parameter estimation scripts in https://github.com/raphael-group/hotnet2 to determine δ and β. As HotNet2 takes in heat scores as its input, we compute them from the p-values using the rule $p\mapsto -{log}_{10}(p)$. Because HotNet2 is very expensive to run, we pre-compute the network files. Modules of size smaller than 3 were discarded.

#### FDRnet:

FDRnet depends primarily on the FDR bound parameter B. Since EMP requires the algorithm to be run on a large number of times on the permuted datasets, we modify it to be more efficient. Specifically, we give FDRnet $B_{1}$ and $B_{2}$ parameters. Seeds are genes with an FDR less than $B_{1}$, and the subnetwork optimization problem instead requires that the subnetwork ${𝒢}_{s}^{*}$ be such that

$$\frac{1}{V\left({𝒢}_{s}^{*}\right)}\sum_{v\in V\left({𝒢}_{s}^{*}\right)}fdr(v)<B_{2}.$$

$B_{1}$ is chosen to be smaller than $B_{2}$ so that seeds of a higher FDR are not chosen in the subnetwork constraint problem. However, they can still appear in other significant subnetworks. On most datasets, we take $B_{1}=0.1$ and $B_{2}=0.3$. $B_{2}$ is chosen to be quite large because FDRnet tends to produce too few modules to meaningfully compare on more stringent thresholds. Modules of size smaller than 3 were discarded. Code was retrieved from https://github.com/yangle293/FDRnet.

### 4.5 Earth Mover’s Distance

The EMD can naturally be formulated as a linear programming problem, according to its definition. Given a network 𝒢 and two modules $M_{1}$ and $M_{2}$ whose vertices are $\left\{u_{1},\ldots ,u_{n_{1}}\right\}$ and $\left\{v_{1},\ldots ,v_{n_{2}}\right\}$ respectively, we seek to

$$\begin{array}{l} {minimize}_{\left\{c_{ij}\right\}}\;\sum_{i\in \left[n_{1}\right]}\;\sum_{j\in \left[n_{2}\right]}\;dist\left(v_{i},v_{j}\right)\cdot c_{ij}\\ \;\text{subject}\;\text{to}\;\sum_{j\in \left[n_{2}\right]}\;c_{ij}=\frac{1}{n_{2}}\;\forall i\in \left[n_{1}\right]\\ \;\sum_{i\in \left[n_{1}\right]}\;c_{ij}=\frac{1}{n_{1}}\;\forall j\in \left[n_{2}\right]\\ \;c_{ij}⩾0\;\forall i\in \left[n_{1}\right],j\in \left[n_{2}\right] \end{array}$$

where dist(⋅,⋅) is computed by Dijkstra’s algorithm. The optimization variables $c_{ij}$ represent the flow from $u_{i}$ to $v_{j}$. The optimization is solved separately for each pair of modules being compared. <MONO@SPACE>PuLP</MONO@SPACE> (version 2.9.0) for Python 3.10.2 was used to solve the optimization problem.

### 4.6 Spectral Clustering

Given the gene-gene coocurence matrix C, we define the degree matrix D as a diagonal matrix whose entries are

$$D_{ii}=\sum_{j=1}^{n}C_{ij}.$$

We note that C is a symmetric matrix. We then perform spectral clustering on the normalized Laplacian

$$L=I-D^{-1/2}CD^{-1/2}.$$

We estimate the number of clusters using the eigengap heuristic. Specifically, let $0=\lambda_{1}⩽\lambda_{2}⩽\cdots ⩽\lambda_{n}$ be the eigenvalues of L arranged in nondecreasing order. We set the number of clusters to be

$$k^{*}\;:=\underset{2⩽i⩽n-1}{argmax}\left|\lambda_{i}-\lambda_{i+1}\right|.$$

**Figure 11:**
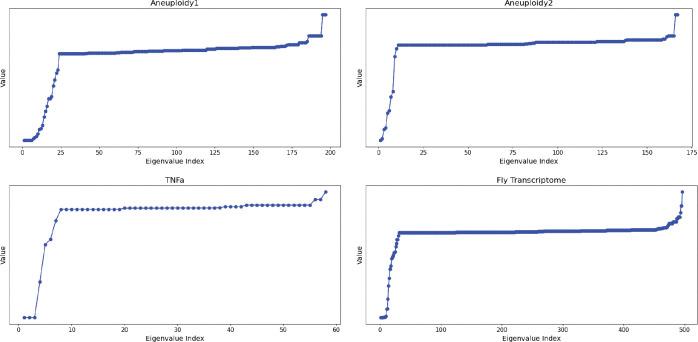
**Eigenvalues of the normalized Laplacian for each dataset.** We choose the number of clusters to be the index of the eigenvalue that maximizes the difference in consecutive eigenvalues. We set $k^{*}=23,\;8,\;4,\;13$ for Aneuploidy1, Aneuploidy2, TNFa, and Fly Transcriptome, respectively.

A visualization of the eigenvectors in each dataset is shown in Figure 11. We obtain the clustering as follows: let $v_{1},\ldots ,v_{k^{*}}$ be the eigenvectors of L corresponding to $\lambda_{1},\ldots ,\lambda_{k^{*}}$. Let $U=\left[\begin{array}{lll} v_{1} & v_{2}\cdots & v_{k^{*}} \end{array}\right]\in R^{n\times k^{*}}$ be the matrix whose columns are the eigenvectors. The row-normalized matrix T is given by

$$T_{ij}=\frac{1}{U_{ij}}\sqrt{\sum_{j=1}^{k^{*}}\;U_{ij}^{2}}.$$

Viewing each row in T as an element of $R^{k^{*}}$, we apply k-means with default parameters, which selects initial cluster centers to be distant from each other, and runs the algorithm for a maximum of 300 iterations or until convergence within a tolerance of 10^−4^. We used <mono@space>scikit-learn</mono@space> (version 1.5.1) for Python 3.10.2.

### 4.7 GCM

We provide a detailed description of the GCM algorithm in Algorithm 1.

## Supplementary Material

Supplement 1

Supplement 2

Supplement 3

6 Additional Files

Additional File 1: Detailed description of AMI algorithms.

Additional File 2: Table S1. Complete listing of all modules identified by each algorithm across all datasets.

Additional File 3: Table S2. Merged modules in Aneuploidy1, Aneuploidy2, TNFa, Fly Transcriptome datasets.

## Figures and Tables

**Table 1:** Datasets

Dataset	Technology	Total genes	Significant genes (p-value < 0.05)
Aneuploidy1	WES	18,327	401
Aneuploidy2	WES	18,976	374
TNFa	RNA-seq	13,478	823
Fly Transcriptome	Expression array	12,510	1,005

**Table 2:** Network characteristics

Network	Number of genes/nodes	Number of interactions/edges	Datasets used
SGC	18,113	604,629	Aneuploidy1, Aneuploidy2
STRING	6,708	98,616	Fly Transcriptome
DIP	3,054	5,024	TNFa

**Table 3:** Empirically-Validated to Hypergeometric Ratio (EHR) values.

	Aneuploidy1	Aneuploidy2	TNFa	Fly Transcriptome
PAPER	**0.938 (15/16)**	0.83 (5/6)	**1 (50/50)**	**1 (262/262)**
DOMINO	0.504 (113/224)	0.790 (147/186)	0.987 (239/242)	0.857 (402/469)
HotNet2	0 (0/903)	0.006 (9/1501)	0.988 (165/167)	0.021 (20/923)
FDRnet	0.8 (24/30)	**0.875 (7/8)**	**1 (35/35)**	0.377 (17/45)

EHR value for each algorithm and dataset are followed by the number of EV and HG terms. The highest EHR values for each dataset is bolded.

**Table 4:** Biological Richness.

	Aneuploidy1	Aneuploidy2	TNFa	Fly Transcriptome
PAPER	9 (15)	4 (5)	24 (50)	49 (262)
DOMINO	32 (224)	34 (180)	58 (242)	92 (469)
HotNet2	0 (4)	8 (9)	39 (165)	11 (20)
FDRnet	11 (24)	5 (7)	16 (35)	7 (17)

Richness for each algorithm and dataset are followed by the number of EV terms.

**Table 5:** Intra-module homogeneity

	Aneuploidy1	Aneuploidy2	TNFa	Fly Transcriptome
PAPER	**3.445**	0.333	3.217	3.022
DOMINO	2.241	**2.082**	2.787	**3.365**
HotNet2	0.000	0.189	**3.959**	0.396
FDRnet	1.199	0.500	2.153	2.202

## Data Availability

All datasets used in this study are either publicly available or can be accessed through the repositories listed in the Methods section. Code is available at https://github.com/LiuJ0/AMI-Benchmark.
